# Mobilisation of Data From Natural History Collections Can Increase the Quality and Coverage of Biodiversity Information

**DOI:** 10.1002/ece3.71139

**Published:** 2025-04-13

**Authors:** Bryony Blades, Cristina Ronquillo, Joaquín Hortal

**Affiliations:** ^1^ Department of Biology University of Oxford Oxford UK; ^2^ African Natural History Research Trust (ANHRT) Kingsland Herefordshire UK; ^3^ Department of Biogeography & Global Change Museo Nacional de Ciencias Naturales (MNCN‐CSIC) Madrid Spain

**Keywords:** biodiversity, data coverage, digitisation, GBIF, mobilisation, natural history collections

## Abstract

The surge of biodiversity data availability in recent decades has allowed researchers to ask questions on previously unthinkable scales, but knowledge gaps still remain. In this study, we aim to quantify potential gains to insect data on the Global Biodiversity Information Facility (GBIF) through further digitisation of natural history collections, assess to what degree this would fill biases in spatial and environmental record coverage, and deepen understanding of environmental bias with regard to climate rarity. Using mainland Afrotropical records for *Catharsius* Hope, 1837 (Coleoptera: Scarabaeidae), we compared inventory completeness of GBIF data to a dataset which combined these with records from a recent taxonomic revision. We analysed how this improved dataset reduced regional and environmental bias in the distribution of occurrence records using an approach that identifies well‐surveyed spatial units of 100 × 100km as well as emerging techniques to classify rarity of climates. We found that the number of cells for which inventory completeness could be calculated, as well as coverage of climate types by ‘well‐sampled’ cells, increased threefold when using the combined set compared to the GBIF set. Improvements to sampling in Central and Western Africa were particularly striking, and coverage of rare climates was similarly improved, as not a single well‐sampled cell from the GBIF data alone occurred in the rarest climate types. These findings support existing literature that suggests data gaps on GBIF are still pervasive, especially for insects and in the tropics, and so, is not yet ready to serve as a standalone data source for all taxa. However, we show that natural history collections hold the necessary information to fill many of these gaps, and their further digitisation should be a priority.

## Graphical abstract

Data gaps on the Global Biodiversity Information Facility (GBIF) are well described for the tropics and invertebrates, and here, we show that biases in spatial coverage translate to undersampling of environmental conditions and rare climates. However, our results underline that further mobilisation of natural history collections has the capacity to lessen these gaps, indicating that some of the data required to combat the Wallacean shortfall likely already exists in museums and private collections.

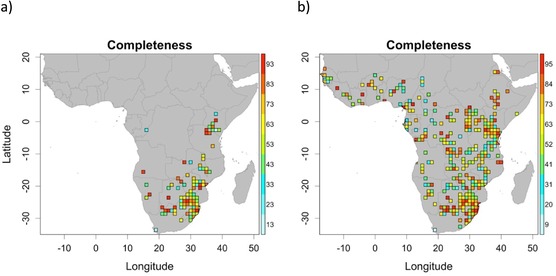

## 1 Introduction

In recent decades, the concurrent intensification of technological advancement and the coupled climate and biodiversity crisis has given rise to massive mobilisation of biodiversity ‘big data’ (Newbold 2010; Wüest et al. 2020). This surge in data availability has allowed researchers to ask questions on previously impossible scales in fields such as conservation, biodiversity informatics, macroecology, disease biology and taxonomy (Heberling et al. 2021). Understanding of species' distributions and methods with which to model them have particularly benefitted, in turn advancing knowledge of evolutionary processes and conservation management (Acevedo et al. 2016; Elith and Franklin 2013; Guisan and Thuiller 2005; Soberón and Peterson 2004).

Despite the scale of these advancements, our understanding of species' distributions is still incomplete. Labelled the ‘Wallacean shortfall’ (Lomolino 2004), knowledge gaps arising from uneven sampling effort result in biases in spatial, temporal, climatic and taxonomic coverage of records (Collen et al. 2008; Hortal et al. 2015; Oliver et al. 2021; Sporbert et al. 2019; Troudet et al. 2017). Despite their richness, data deficits are particularly pervasive in the tropics, driven by tough environmental conditions, limited accessibility, poor infrastructure or security, and a lack of local capacity such as academic institutions or funds (Amano and Sutherland 2013; Araujo et al. 2022; Rocha‐Ortega et al. 2021; Siddig 2019; Yesson et al. 2007). Such record unevenness in space can also result in their biased distribution across environmental gradients, affecting our ability to characterise the real breadth of species' fundamental niches (Hortal et al. 2008)—although this is not always the case (Newbold 2010). This hampers the reliability of model predictions, potentially misinforming applications in ecology and conservation (Troia and McManamay 2016).

Deficits and spatial biases are especially critical for invertebrates (Rocha‐Ortega et al. 2021; Troudet et al. 2017), the importance of which to global biodiversity and ecosystem services cannot be understated (Noriega et al. 2018; Wagner et al. 2021). This has led to particularly low inventory completeness—how comprehensively biodiversity has been surveyed and recorded—even in scenarios where records are numerous (García‐Rosello et al. 2023; Iannella et al. 2019; Sánchez‐Fernández et al. 2021), and that an unknown number may have already gone extinct (García‐Rosello et al. 2023) paints a concerning picture for biodiversity moving forward. Although the strength of the Global Biodiversity Information Facility (GBIF)—the world's largest online biodiversity information network—lies in its compilation of data from myriad sources, these gaps remain, and not yet mobilised (digitised and made widely available through an online database) data sources such as taxonomic bibliographies and natural history collections can still provide novel insights (Beck et al. 2013; Shirey et al. 2019). As such, efforts to investigate spatial and environmental bias in insect sampling generally compile exhaustive databases from a number of sources (Ballesteros‐Mejia et al. 2013; Romo et al. 2006; Sánchez‐Fernández et al. 2008, 2022; Shirey et al. 2021), and less is known about insect inventories derived from GBIF data alone (see García‐Rosello et al. 2023; Girardello et al. 2019; Rocha‐Ortega et al. 2021; Troia and McManamay 2016). Additionally, whether inventory completeness is biased towards climate conditions that occur frequently is seldom analysed (but, see Ronquillo et al. 2020; Sobral‐Souza et al. 2021), despite evidence that GBIF data are lacking in rare, locally restricted taxa (Beck et al. 2013).

Given the prevalence of correlative species distribution modelling, a technique that assumes comprehensive sampling of a species' fundamental niche using GBIF data (Heberling et al. 2021), it is problematic that research to date has not yet fully determined how significantly the completeness of its insect inventories is biased in climatic space. The strength of GBIF, though, lies in its role as a centralised aggregator, and its capacity as a collaborative platform can be leveraged to reduce this bias. Intensified digitisation and mobilisation of natural history collections have begun to help, especially with contributions from smaller herbaria and institutions, as well as private collections (Araujo et al. 2022; Beck et al. 2013; Yesson et al. 2007), but much remains to be done (Hardy et al. 2023; Popov et al. 2021). Fortunately, data compilation for purposes such as taxonomic revision often utilises collections that have heretofore not been mobilised, allowing an examination of how additional data could improve inventory completeness across space and climate and, here, for a severely data‐deficient clade.

In this study, spatial and environmental bias in GBIF insect inventories is evaluated using data on dung beetles, which are known to act as a proxy for general biodiversity (Spector 2006). We determine how significantly bias could be reduced through the integration into GBIF of a dataset independently compiled for a taxonomic revision of the Afrotropical members of the genus *Catharsius* Hope, 1837 (Coleoptera: Scarabaeidae) (Takano 2025). In particular, we investigate inventory completeness and how evenly this is distributed across climatic conditions and rarity.

## 2 Materials and Methods

### 2.1 Study Area and Taxon

The study area is the African mainland of the Afrotropical biogeographical realm spanning 17.5°W–51°E, 21°N–35°S. The study region exhibits a wide breadth of climatic conditions, including eight biomes and 91 ecoregions, encompassing tropical forest to xeric shrublands. Using QGIS version 3.22.9‐Białowieża (QGIS 2021), a shapefile of the realm (Olson et al. 2001; World Wildlife Fund 2012) was modified to include only the mainland of Africa, categorising countries by region using the UN M49 standard (United Nations Statistics Division n.d.) as a reference (see Appendix S1). This shapefile was then used to create a grid of 100 km × 100 km cells for the statistical analyses, a resolution chosen given the scale of the study region and general sparsity of GBIF *Catharsius* records.

**FIGURE 1:**
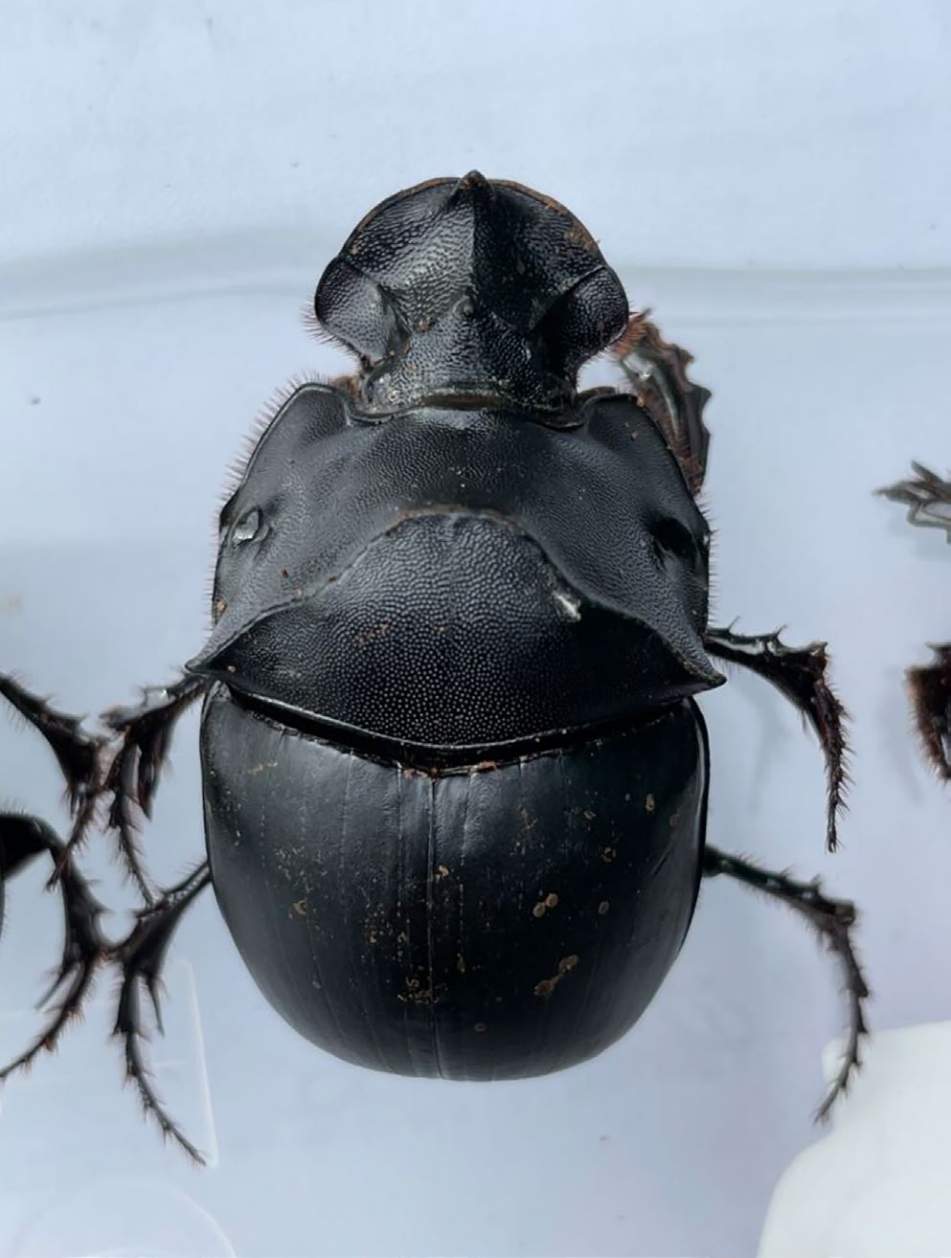
An Afrotropical member of *Catharsius, C* *. dux* , in NW Zambia (2022).

*Catharsius* is a genus of large copro‐ and necrophagous dung beetles with species distributed across both Africa and Asia. The Afrotropical members, including *Catharsius dux* (Figure 1), are currently being revised in the largest ever revision of any group of dung beetles in the world, for which an extensive collection of distributional data has been compiled from natural history collections (Takano 2025). It is these records that have been collated (Blades and Takano 2024) and are described below.

### 2.2 Occurrence Data

To demonstrate the potential value of integrating unmobilised natural history collection data to GBIF, two datasets were used. First, all occurrences for *Catharsius* were downloaded from GBIF (2023) and subjected to a preprocessing procedure to ensure they did not fall foul of known GBIF data quality issues, as follows. Occurrences were filtered to include only those identified to the species level, and with precise and accurate coordinate information that did not suggest specimens were located in biodiversity institutions, the sea, in the centroid of a country, or in countries other than those listed on the physical label. The taxonomy of the resulting occurrences was standardised according to the most recent revision (Takano 2025) by removing occurrences that were identified as a non‐African species and those that had been placed into *incertae sedis*, and correcting identifications to reflect new synonyms and homonyms. Further specifics of these processes can be found in Appendix S2. The geographic distribution of the resulting occurrences was cropped to the same extent as the bioclimatic variables, and this is henceforth referred to as the ‘GBIF set’.

Second, a combined dataset was created by joining the GBIF set and a set of species names, coordinates, years and counts that had previously been extracted from the text of the taxonomic revision—the ‘revision set’ (Blades and Takano 2024). As part of the extraction process, all occurrences had been manually inspected to ensure they fell on land and in countries that were expected for that species. A small number of errors from typing inaccuracies were corrected with the expert help of the original taxonomist, and all records were cropped to the study extent before merging with GBIF data. Some of the records had been mobilised on GBIF, likely due to varying data sharing agreements between institutions holding the specimens, so duplicate entries were then manually removed. To be considered a duplicate, records must have shared the same species name and year, and in the circumstance that a more precise date was listed in either set, this must be identical. Records must also have had the same collector and location names, even if these were written in different formats, and neither their latitude nor longitude could be more than one geographic degree apart. Further specifics can be found in Appendix S2. This is henceforth referred to as the ‘combined set’.

### 2.3 Statistical Analyses

To assess survey completeness in all grid cells of the study area, the package ‘KnowBR’ version 2.2 (Guisande and Lobo 2023) was used. By generating species accumulation curves—representing the cumulative number of species observed as a function of the cumulative number of samples collected—it compares the number of observed species to the number of predicted species per spatial unit (here, the 100 km × 100 km cells). This determines a percentage of inventory completeness, that is, how many of the species that are likely to be present in each cell have been recorded as such (Lobo et al. 2018). Analysis was carried out separately for both the GBIF and combined sets, and then cells with > 20 records, a completeness score > 75% and a ratio of occurrences to the number of species > 5 were identified as ‘well‐sampled’ (WS) in each set. These criteria used the approximate plateau of the cumulative distribution of records within each measure as a reference (see Ronquillo et al. 2023). The regional shapefile was used to quantify the bias of inventory completeness and WS cells for both sets in terms of how evenly they were distributed in each region.

As unevenness in the spatial coverage of occurrence data has been shown to result in biased sampling of environmental conditions (Hortal et al. 2008), the WS cells for each set were then used to evaluate if comprehensive sampling has been conducted across the full spectrum of potential environmental conditions in the study region. To describe environmental variations in the study area, we used the 19 bioclimatic variables from WorldClim, which represent trends, seasonality and limiting environmental factors of temperature and precipitation derived from aggregating monthly data from the period 1970–2000 (Fick and Hijmans 2017; WorldClim 2020; see Appendix S3). These were downloaded at a resolution of 30 s (approximately 1 km at the equator) and aggregated to 0.83° (approximately 100 km at the equator), the resolution of the grid used for spatial coverage, and cropped to the study extent.

**FIGURE 2:**
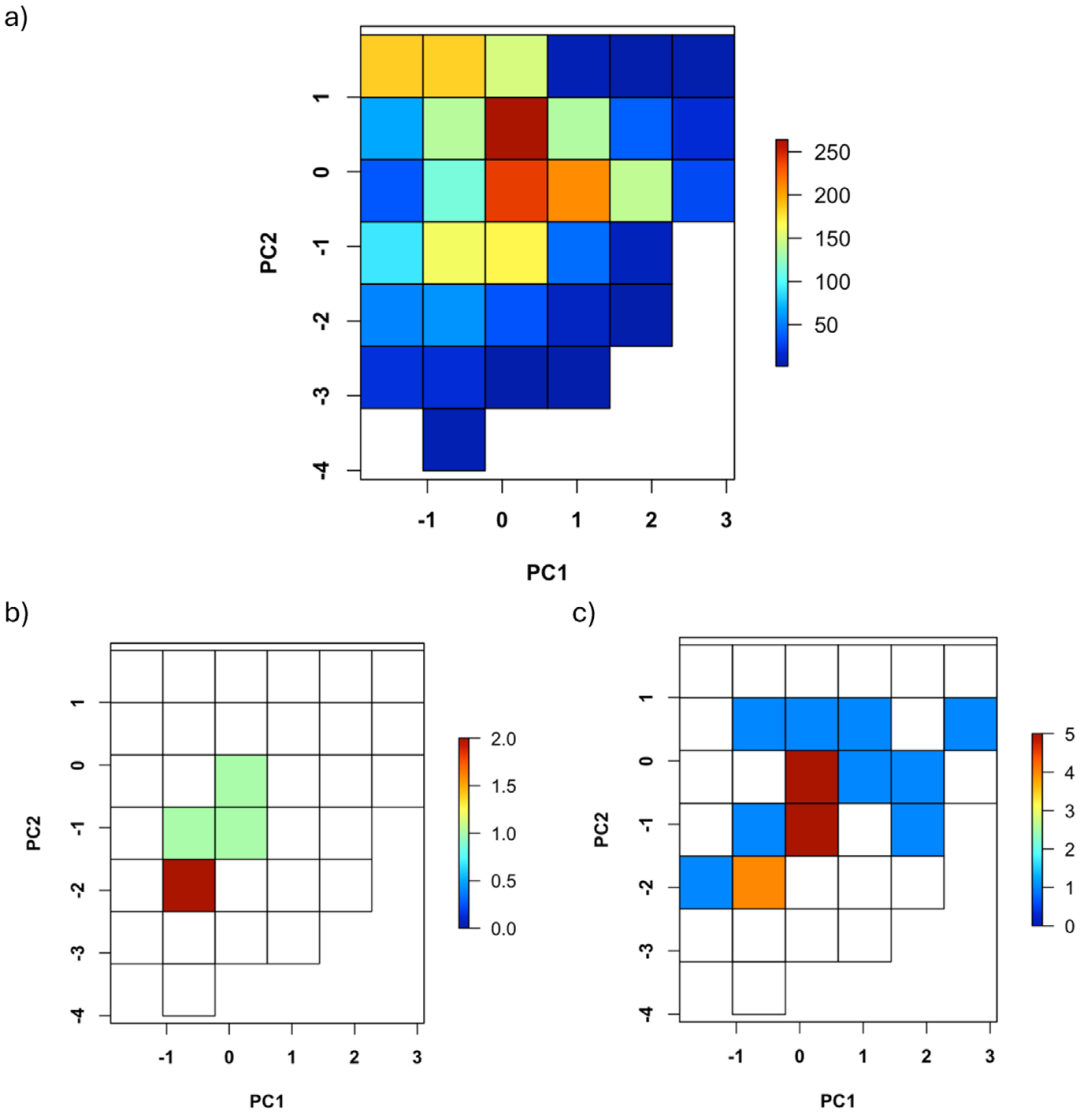
Thirty‐three climate types were identified in the Afrotropical mainland when principal components analysis values were converted into classes (a). Climate types found in well‐sampled *Catharsius* cells for the GBIF set (b) and combined set (c) are displayed with colour indicating the number of cells that particular climate type was found in.

A principal components analysis (PCA) of the study area was carried out in R package ‘psych’ version 2.4.1 (Revelle 2024) to reduce the dimensionality of the climate data to two axes (PC1 and PC2) that captured 73% of the variation. PC1 predominantly characterised levels of precipitation and variability in temperature, displaying a gradient from the high rainfall and more stable temperatures of tropical forests to the drier desert areas with greater annual and daily temperature ranges. PC2 broadly described a temperature gradient from the warmer north and low elevations to the cooler south and high elevations, as well as high to low precipitation seasonality to a lesser degree. Then, these axes were converted into classes by binning the PCA environmental space into equal‐area cells (see Sobral‐Souza et al. (2021) and Ronquillo et al. (2020) for a similar approach). The resulting bins correspond to distinct ‘climate types’ of which 33 were identified in this study area (Figure 2a). Although other approaches may provide a more continuous definition of climate rarity (see Fournier et al. 2020), the method used here has the advantage of ensuring that the analysis of WS cells uses the exact same description of climate frequency in both coverage of general climate conditions and climatic rarity.

The classes—or ‘climate types’—and the frequency at which each was observed in the study region were quantified. The climate types that the WS cells fell into were identified, and the niche overlap between conditions in these locations and the study region as a whole was calculated using Schoener's *D*. This returns a value of between 0, for no overlap, and 1, for a complete overlap (Schoener 1970). Here, as WS cells are found in an increasing number of climate conditions, Schoener's *D* would be expected to rise, indicating good sampling over a more comprehensive coverage of potential environmental conditions in the Afrotropical realm. The significance of *D* values was tested by comparing them against a null distribution generated by randomly sampling 1000 sets of five (for the GBIF set) or 23 (for the combined set) occurrences (i.e., the same number of occurrences as WS cells for each set) and calculating the niche overlap of each sample with the study area.

To determine whether environmental conditions found in WS cells were an unbiased subset of those described by each PCA axis—that is, whether better sampling is correlated with particular conditions—two‐sample Kolmogorov–Smirnov tests were run using the R base package ‘stats’. Used to evaluate whether two groups are sampled from the same distribution (Massey 1951), the null hypothesis will be rejected if the WS cells sample certain climatic conditions at a rate that does not reflect their overall frequency in the study area. For example, if WS cells are found to disproportionately favour cooler climates, despite the presence of warmer climates in the realm.

Finally, min‐max scaling was used to evaluate whether WS cells were found in climates that occur infrequently in the study region, or are ‘rare’. For this, a rarity value between 0 (common) and 1 (rare) was assigned to each climate type, depending on its relative frequency in the study region. The rate at which each type was sampled by WS cells was then compared to its overall density in the study region, as such determining whether good sampling was biased towards common or rare climates.

All analyses were conducted in R version 4.2.1 (R Core Team 2022), using RStudio version 2023.12.1.402 (Posit Team 2024), and code adapted from Ronquillo (2023).

## 3 Results

### 3.1 Preprocessing of Occurrence Data

Downloading all records for *Catharsius* from GBIF returned a dataset of 4270 entries (GBIF 2023), pertaining to 72 species; more results of the quality assurance filtering and taxonomic standardisation procedures can be found in Appendix S2. After preprocessing, the GBIF set totalled 1686 entries, corresponding to 3915 specimens belonging to 50 species. The revision dataset totalled 4979 entries, corresponding to 15,943 specimens belonging to 146 species. Upon combination with the GBIF dataset, 489 duplicate entries were removed from the former, resulting in a combined dataset of 6174 entries, corresponding to 18,043 specimens belonging to 146 species. As such, the revision set contributed the overwhelming majority of information to the combined set, with GBIF having contributed only 27.3% of entries, 21.7% of specimens and no new species.

### 3.2 Statistical Analyses

**FIGURE 3:**
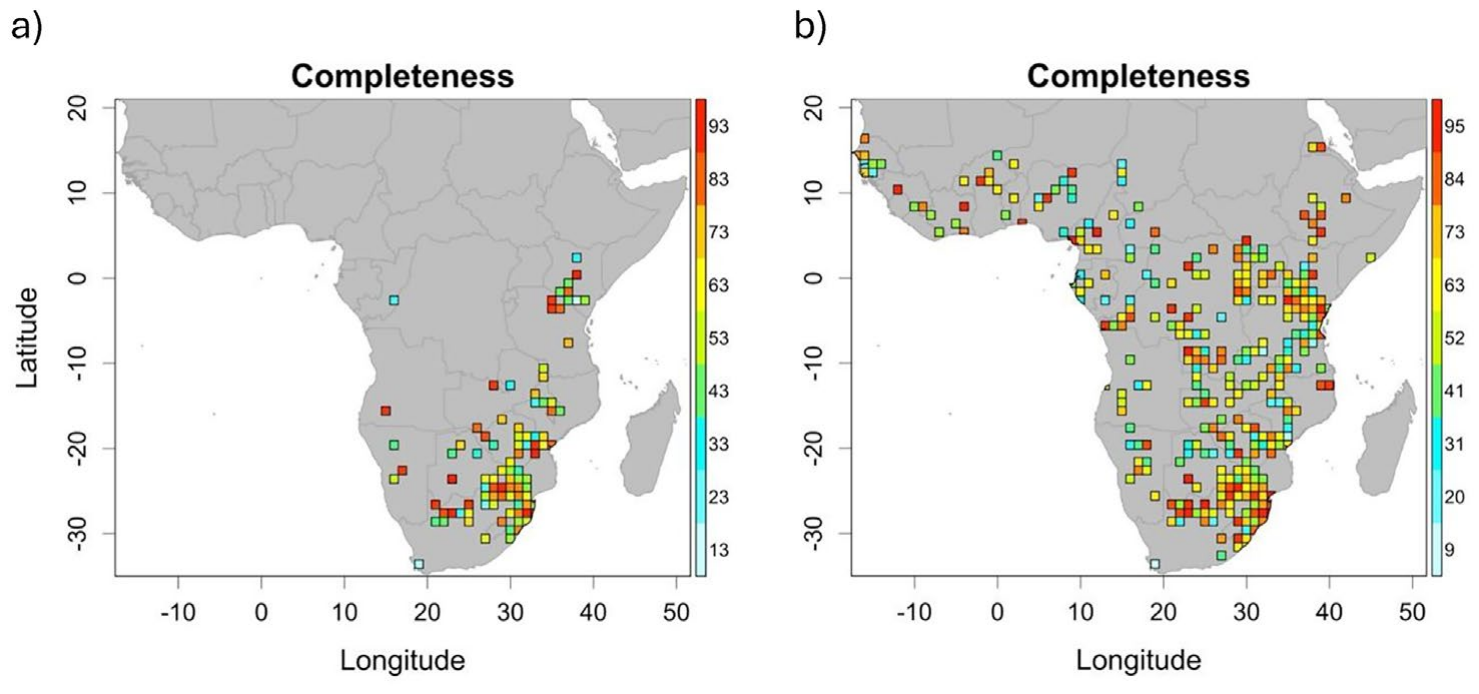
Completeness of *Catharsius* inventories in the mainland Afrotropical realm using the (a) GBIF set and (b) combined set. Coloured cells are those for which data were sufficient to calculate completeness, with warmer colours indicating higher completeness percentage.

A total of 94 cells, out of a potential 1867 (5%), contained sufficient information to compute inventory completeness for the GBIF set, which is predominantly concentrated in the northeast of South Africa (Figure 3). Regionally, 51 of these cells are found in Southern Africa, 41 in Eastern Africa, two in Central Africa and none in Western Africa. Contrastingly, inventory completeness could be computed for 314 (16.82%) with the combined set, an increase of 220. Of these, 75 are found in Southern Africa, 128 in Eastern Africa, 76 in Central Africa and 35 in Western Africa, illustrating a reduced regional sampling bias. Some cells for the combined set lie across the border between two regions, in which case the centre point of the grid square was the decider.

Cells with high completeness values were also concentrated in South Africa, with some further representation in Southern and Eastern Africa for the GBIF set, but a cross‐realm spread for the combined set. The single grid cell in Central Africa with a high completeness value in the GBIF set was no longer considered to be so well completed in the context of a more extensive dataset.

**FIGURE 4:**
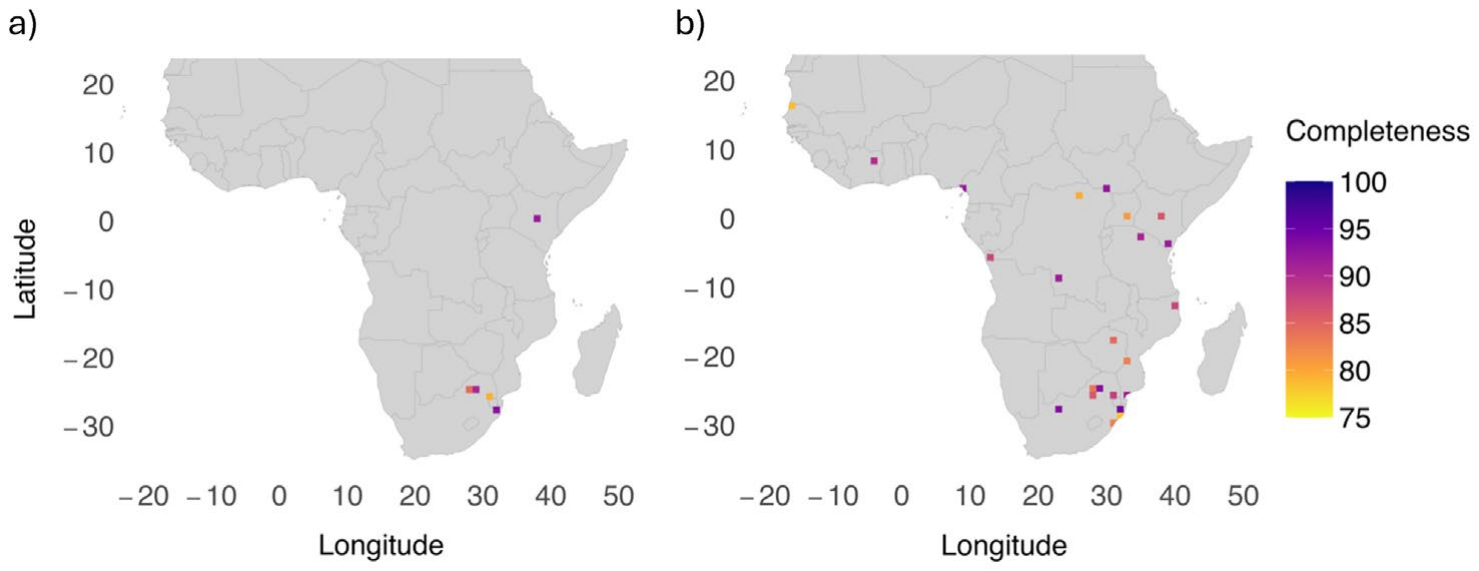
Cells in the mainland Afrotropical realm in which *Catharsius* is well‐sampled using (a) the GBIF set and (b) the combined set, where well‐sampled is defined as containing > 20 records, completeness > 75% and a ratio of occurrences to species > 5. Colour indicates completeness percentage, with darker colours identifying higher completeness.

Only five cells fulfilled the criteria to be considered well‐sampled using the GBIF set, with four in the northeast of South Africa and one in Kenya; just 0.27% of 1867 potential cells and two countries. Using the combined set, 23 WS cells (1.23%) were identified across 11 countries (8 in South Africa, 3 in the Democratic Republic of Congo, 3 in Mozambique, 2 in Kenya and 1 in each of Senegal, Cote d'Ivoire, Cameroon, South Sudan, Zimbabwe, Uganda and Tanzania). Although South Africa is also comparatively overrepresented, and seven out of these 11 countries only returned a single WS cell, the combined set generated 4.5 times more WS cells in 5.5 times more countries than the GBIF set (Figure 4). Only four out of a potential 33 distinct climate types were found in WS cells from the GBIF set (12.12%; Figure 2b), as opposed to 12 for the combined set (36.36%; Figure 2c). The niche overlap (*D*) between WS cells and the study region as a whole was 0.238 (*p* = 1) and 0.468 (*p* = 1) for the GBIF and combined set respectively.

**FIGURE 5:**
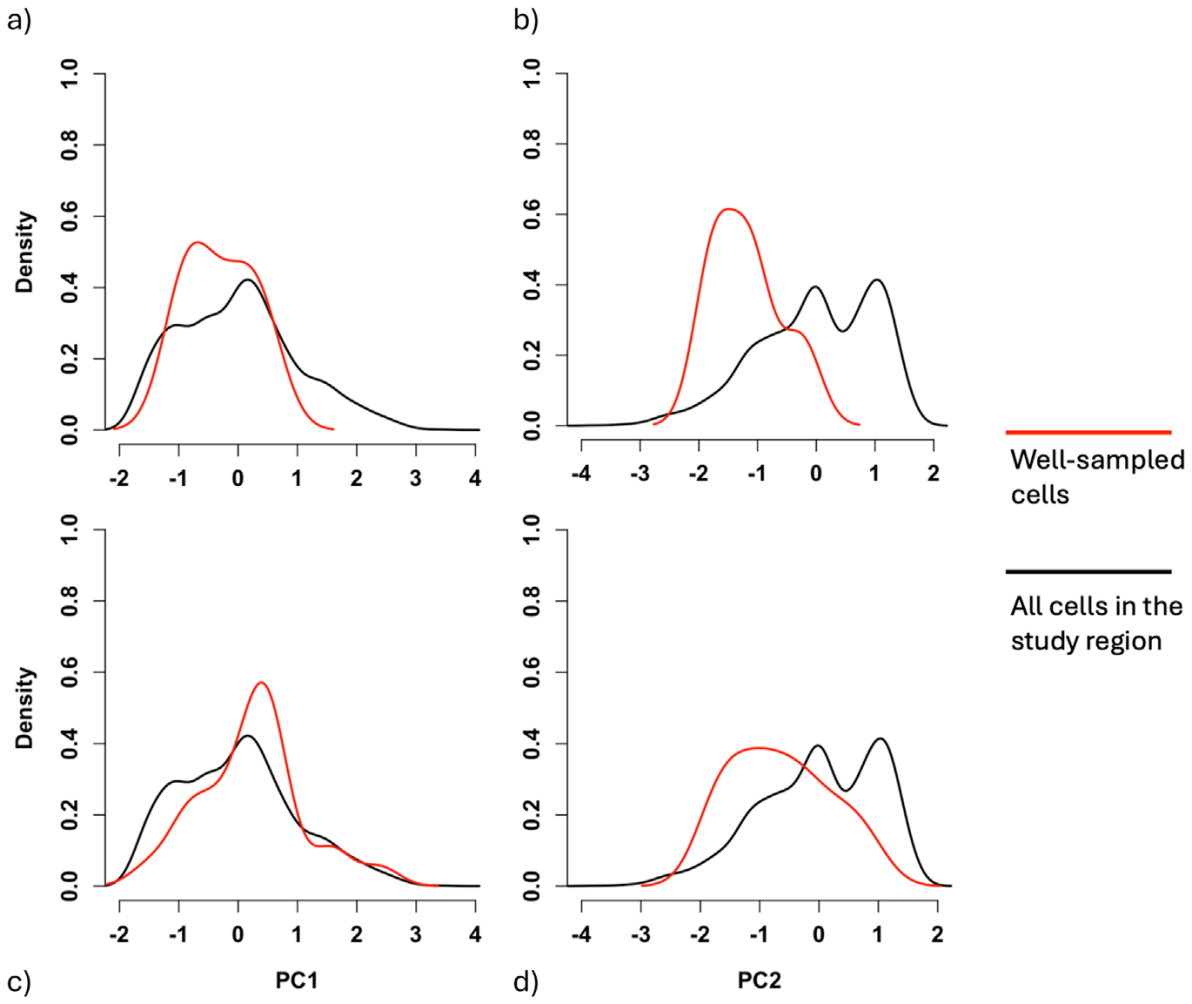
Smoothed kernel density estimate of the distribution of principal component axes (PC1 and PC2) scores for the GBIF set (a and b) and the combined set (c and d), with the y‐axis representing relative density. The red and black lines illustrate the continuous distribution of PCA values found in the *Catharsius* well‐sampled cells and all cells in the mainland Afrotropical realm respectively.

Kolmogorov–Smirnov tests show WS cells for both sets as unbiased in PCA1 (GBIF: *D* = 0.30, *p* = 0.75; combined: *D* = 0.21, *p* = 0.26) but biased in PCA2 (GBIF: *D* = 0.63, *p* = 0.04; combined: *D* = 0.35, *p* = 0.0083). As such, whilst both are representative samples of potential levels of precipitation and temperature variability, they oversample moderate and cooler temperatures, with comparatively little representation of warmer climates in the north (Figure 5).

**FIGURE 6:**
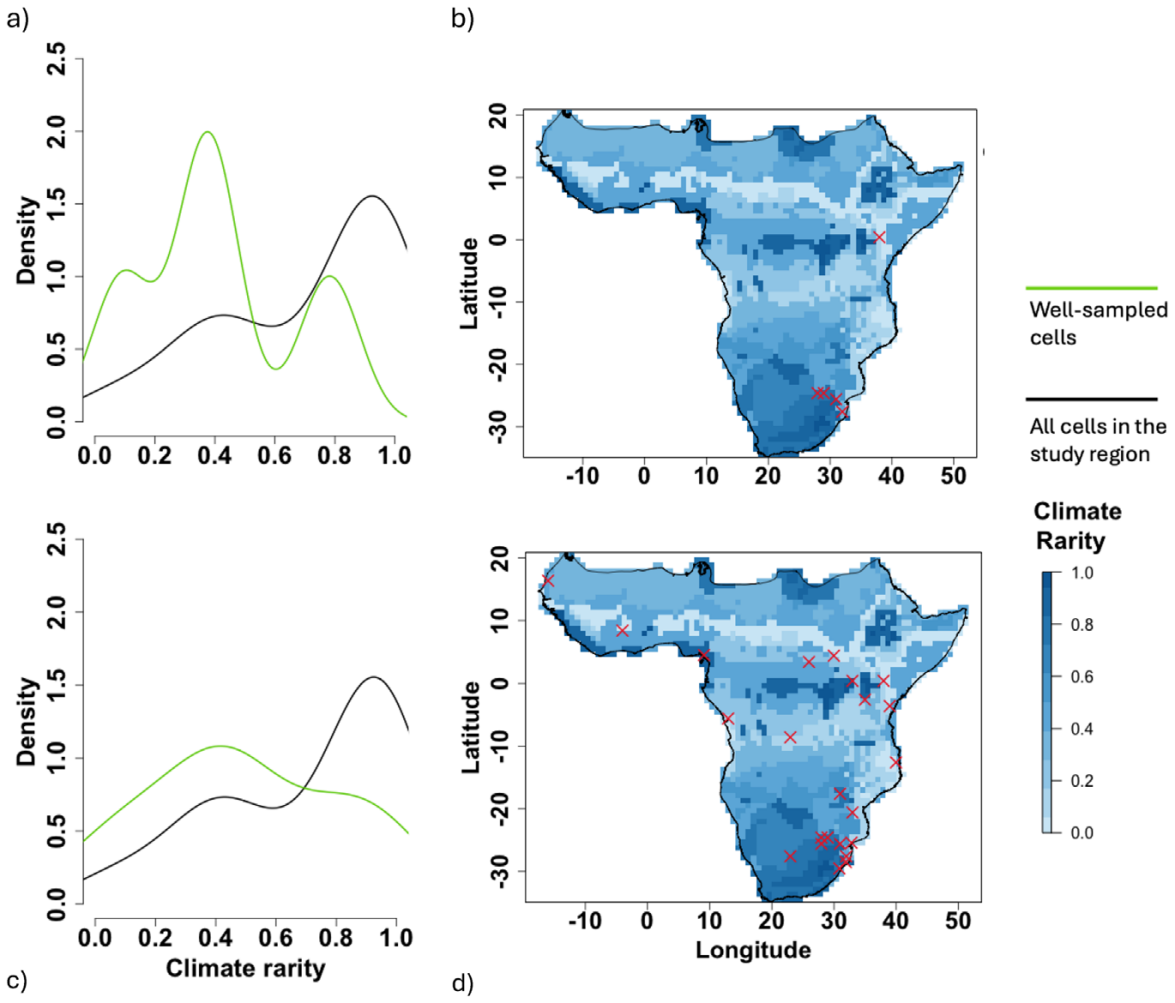
Smoothed kernel density estimate of the distribution of climate rarity scores, between 0 (common) and rare (1), for the GBIF set (a) and the combined set (c), with the y‐axis representing relative density. The green and black lines illustrate the continuous distribution of climate rarity values found in the *Catharsius* well‐sampled cells and all cells in the mainland Afrotropical realm respectively. Maps illustrate the geographic distribution of climate rarity, with dark colours representing rarer climates, and well‐sampled *Catharsius* cells for the GBIF set (b) and the combined set (d).

No WS cells from the GBIF set were found in the rarest of climates (rarity > 0.8), and both very common and moderately common climate types were overrepresented compared to their relative density in the study region. Whilst WS cells from the combined set also undersampled the rarest of climates, it was to a lesser degree, and common and moderately common climate types were sampled at a rate more representative of their relative density in the study region (Figure 6a,c). Improved sampling in Central and Eastern Africa was responsible for coverage of the rarest climates by combined set WS cells, and the failure of GBIF WS cells to sample any of these was despite them both being most prevalent in South Africa (Figure 6b,d).

## 4 Discussion

This study provides novel insights into the inventory completeness of the world's biggest online biodiversity data network. By quantifying the coverage improvement achieved with the addition of further natural history collection records, this comparative approach assesses not just how much value is still missing for insects on GBIF, but also the scale of potential gains from further record digitisation. In particular, it has shown that GBIF occurrence records are biased towards the southern and eastern areas of the Afrotropical realm and, consequently, fail to sample across the full range of potential environmental conditions. This leads to poor coverage of warmer climates and rare climate types. Whilst the inclusion of further natural history collection data does not entirely remove sampling bias, it greatly reduces unevenness in spatial and environmental sampling.

Inventory completeness for GBIF insect data is well documented as being poor worldwide, particularly so outside of Europe (García‐Rosello et al. 2023; Rocha‐Ortega et al. 2021), and this is reflected very clearly in these results. Here, GBIF data are only sufficient to compute completeness for 5% of grid cells and, even then, many of these return poor values and are strongly regionally biased, the pattern of which is broadly comparable with the general inventory completeness of insects on GBIF (Figure 2c, García‐Rosello et al. 2023, 493). The combined set, though, allows completeness to be computed for over three times as many cells, and regional gaps are filled. It provides enough data to compute values in 74 and 35 more cells in Central and Western Africa, respectively, than the GBIF set, and also generated highly completed cells more evenly across the realm.

Notably, the two highly completed GBIF cells in Central Africa intersect Mupa and Bicuari National Parks in Angola, which is consistent with Girardello et al. (2019) who found gaps in GBIF butterfly inventories to correlate with the low density of protected areas. In fact, these results support that geographic biases in GBIF insect inventory completeness are comparable to those in GBIF raw data (García‐Rosello et al. 2023), as they identify deficiencies driven by survey area attractiveness and socioeconomic factors. The GBIF set generated 4.5 times less WS cells than the combined set, and their exclusive location in South Africa is consistent with findings that GBIF sampling coverage is strongly related to GDP per capita (Amano and Sutherland 2013; Hughes et al. 2021; IMF 2024), and shows similarities with the work of Stropp et al. (2016) on seed plants, evidencing that many biases in biodiversity knowledge are pervasive across groups. Contrastingly, almost a third of the combined set WS cells were generated in countries ranked in the bottom 10 for 2024 GDP per capita (Democratic Republic of Congo, Mozambique and South Sudan) (IMF 2024). Species data used in this study are not from 2024, but this does pose the question of whether bias in GBIF data driven by economic inequality could be reduced by further digitisation of natural history collections. Critically, records are made available on GBIF through data‐sharing agreements between the platform and contributing institutions, and this study indicates that further mobilisation should leverage GBIF's strength as a centralised aggregator and aim to expand its pool of contributors rather than just the volume of information from existing participants.

In light of the stark regional biases in GBIF WS cells, it is not surprising that coverage of climatic conditions also falls short. Well‐sampled cells from the combined set covered more climate types and had a higher niche overlap with the overall study region, demonstrating the potential value added to ecological inferences through increased record digitisation. That said, nonsignificant D values for both the GBIF set and the combined set indicate that WS cells are predominantly found in common climate types for both, despite general coverage improvement through the integration of further data. These results align with studies in which systematic surveys and natural history collections outperformed GBIF insect data in terms of survey coverage, but also derivation of ranges and climatic niches (Beck et al. 2013; Troia and McManamay 2016). It is concerning that the scale of bias in GBIF insect data is such that it compromises its usefulness despite the sheer number of records now accessible. This has also been observed in other taxa (Araujo et al. 2022; Shirey et al. 2019), and whilst its severity is likely not consistent across groups, it is probable that GBIF is not yet comprehensive enough to be used as an exclusive data source in modelling biodiversity patterns and should instead be used as a resource within a wider suite. This highlights the importance of investing in collection, digitisation and mobilisation of further datasets through the platform, as improving its coverage would reduce the need to source data from multiple, sometimes inaccessible, or nonstandardised datasets. Others have also called for the integration of biodiversity databases in one place to this same end (Araujo et al. 2022).

A further limitation of GBIF data here was that, whilst most climate types in this study occurred infrequently, this was not reflected in sampling; the GBIF WS cells did not occur in any of the rarest climate types, despite both being most prevalent in South Africa. This risks missing species that may be specialised to infrequent climatic conditions and, in the case of such habitat specificity, at risk on multiple fronts: smaller geographic range, few populations and specialised niche conditions (Işik 2011). It is intuitive that mobilising more records on GBIF increases coverage and combats these weaknesses. Whilst not necessarily a given, as unmobilised specimens in natural history collections may well have been collected in the same places as existing online records, multiplying the quantity of data available is likely to provide new information, especially for a data‐deficient group, as seen above. However, the relative density of the combined set WS cells across climate types and rarities (i.e., the likelihood of their being found in any given place) more closely resembles that of all cells in the study region, making it clear that this is not simply a case of increasing available data. The changing shapes of the smoother kernel density estimate curves in Figures 5 and 6 are a visual representation of closing climatic gaps in sampling. Explicitly, the data from the taxonomic revision are distributed differently across space and environment from those already available on GBIF, and aggregating these fills knowledge gaps.

Usefulness of digitised records, though, hinges on the quality of the data that is generated. Here, over half of the original GBIF data were removed during preprocessing as they did not meet the necessary standards for analysis (see Appendix S2), and concerns with geospatial errors, insufficient metadata and taxonomic inaccuracies are well documented (Ferro and Flick 2015; Prudic et al. 2023; Rocha‐Ortega et al. 2021; Ronquillo et al. 2020; Yesson et al. 2007). Misidentifications and poor taxonomy are particularly difficult to pinpoint and correct after mobilisation (Soberón et al. 2002), and this is especially so for little‐known or cryptic invertebrate species, such as those in *Catharsius*. The revision set data were extracted from a rigorous, multiyear project to revise *Catharsius*' taxonomy, so there were very few georeferencing errors and no taxonomic errors, according to this most recent understanding of the genus. The time‐consuming nature of this work and declining taxonomic expertise (Hopkins and Freckleton 2002; Hutchings 2021; ‘Importance of Taxonomy’ 1946; Lagomarsino and Frost 2020; Wägele et al. 2011) precludes this as an option for many studies, especially those that encompass thousands or even millions of occurrence records. Methods to reduce GBIF taxonomic misidentifications have been tested with some success (Smith et al. 2016), but these still have distinct time and data demands of their own. Other tools to validate not just the taxonomic, but also geographic, temporal and metadata accuracy of records are also promising (Ronquillo et al. 2024), but the quality of GBIF data is such that these processes often greatly reduce the number of usable data points, as seen in this study. It seems clear that the best way to avoid compounding existing flaws in data quality is with meticulous digitisation in the first place, including the allocation of resources to taxonomic verification or revision as part of the data preparation process.

Efforts to understand the limitations of our knowledge are paramount. Research that seeks to better understand these flaws and suggest solutions not only improves theoretical knowledge of species distributions but can potentially better inform practical applications, such as directing fieldwork or prompting new data‐sharing agreements with institutions whose data will maximise biogeographical inference. This study underlines that much value still stands to be gained by further digitisation of natural history collections, emphasising that GBIF is not yet ready to function as a standalone data source, but care must be taken to not compound existing data quality issues.

## Author Contributions

**Bryony Blades:** conceptualization (equal), data curation (lead), formal analysis (lead), methodology (equal), project administration (lead), validation (equal), visualization (equal), writing – original draft (lead), writing – review and editing (equal). **Cristina Ronquillo:** conceptualization (supporting), data curation (supporting), formal analysis (supporting), methodology (supporting), validation (equal), visualization (equal), writing – review and editing (supporting). **Joaquín Hortal:** conceptualization (equal), data curation (supporting), formal analysis (supporting), methodology (equal), supervision (lead), validation (equal), visualization (supporting), writing – review and editing (supporting).

## Conflicts of Interest

The authors declare no Conflicts of Interest.

## Supporting information


Data S1.

## Data Availability

Data and code supporting this paper are cited in this manuscript as (Blades & Takano, 2024), and are available at: 10.25446/oxford.27195816.

## References

[ece371139-bib-0001] Acevedo, P. , A. Jiménez‐Valverde , P. Aragón , and A. Niamir . 2016. “New Developments in the Study of Species Distribution.” In Current Trends in Wildlife Research, edited by R. Mateo , B. Arroyo , and J. T. Garcia , 151–175. Springer International Publishing. 10.1007/978-3-319-27912-1_7.

[ece371139-bib-0002] Amano, T. , and W. J. Sutherland . 2013. “Four Barriers to the Global Understanding of Biodiversity Conservation: Wealth, Language, Geographical Location and Security.” Proceedings of the Royal Society B: Biological Sciences 280, no. 1756: 2649. 10.1098/rspb.2012.2649. PMC3574366 23390102

[ece371139-bib-0003] Araujo, M. L. , A. C. Quaresma , and F. N. Ramos . 2022. “GBIF Information Is Not Enough: National Database Improves the Inventory Completeness of Amazonian Epiphytes.” Biodiversity and Conservation 31, no. 11: 2797–2815. 10.1007/s10531-022-02458-x.

[ece371139-bib-0004] Ballesteros‐Mejia, L. , I. J. Kitching , W. Jetz , P. Nagel , and J. Beck . 2013. “Mapping the Biodiversity of Tropical Insects: Species Richness and Inventory Completeness of A Frican Sphingid Moths.” Global Ecology and Biogeography 22, no. 5: 586–595. 10.1111/geb.12039.

[ece371139-bib-0005] Beck, J. , L. Ballesteros‐Mejia , P. Nagel , and I. J. Kitching . 2013. “Online solutions and the ‘Wallacean shortfall’: What does GBIF contribute to our knowledge of species' ranges?” Diversity and Distributions 19, no. 8: 1043–1050. 10.1111/ddi.12083.

[ece371139-bib-0006] Blades, B. , and H. Takano . 2024. Catharsius Inventory Completeness [Dataset]. 195816. Oxford. 10.25446/oxford.27195816.

[ece371139-bib-0007] Collen, B. , M. Ram , T. Zamin , and L. McRae . 2008. “The Tropical Biodiversity Data Gap: Addressing Disparity in Global Monitoring.” Tropical Conservation Science 1, no. 2: 75–88. 10.1177/194008290800100202.

[ece371139-bib-0008] Elith, J. , and J. Franklin . 2013. “Species Distribution Modeling.” In Encyclopedia of Biodiversity, 692–705. Elsevier. 10.1016/B978-0-12-384719-5.00318-X.

[ece371139-bib-0009] Ferro, M. L. , and A. J. Flick . 2015. “Collection Bias and the Importance of Natural History Collections in Species Habitat Modeling: A Case Study Using *Thoracophorus Costalis* Erichson (Coleoptera: Staphylinidae: Osoriinae), with a Critique of GBIF.Org.” Coleopterists Bulletin 69, no. 3: 415–425. 10.1649/0010-065X-69.3.415.

[ece371139-bib-0010] Fick, S. E. , and R. J. Hijmans . 2017. “WorldClim 2: New 1‐Km Spatial Resolution Climate Surfaces for Global Land Areas.” International Journal of Climatology 37, no. 12: 4302–4315. 10.1002/joc.5086.

[ece371139-bib-0011] Fournier, B. , H. Vázquez‐Rivera , S. Clappe , L. Donelle , P. H. P. Braga , and P. R. Peres‐Neto . 2020. “The Spatial Frequency of Climatic Conditions Affects Niche Composition and Functional Diversity of Species Assemblages: The Case of Angiosperms.” Ecology Letters 23, no. 2: 254–264. 10.1111/ele.13425. 31749270

[ece371139-bib-0012] García‐Rosello, E. , J. Gonzalez‐Dacosta , C. Guisande , and J. M. Lobo . 2023. “GBIF Falls Short of Providing a Representative Picture of the Global Distribution of Insects.” Systematic Entomology 48, no. 4: 489–497. 10.1111/syen.12589.

[ece371139-bib-0013] GBIF . 2023. “Occurrence Download [Dataset].” 10.15468/DL.73MEZG.Global Biodiversity Information Facility.

[ece371139-bib-0014] Girardello, M. , A. Chapman , R. Dennis , L. Kaila , P. A. V. Borges , and A. Santangeli . 2019. “Gaps in Butterfly Inventory Data: A Global Analysis.” Biological Conservation 236: 289–295. 10.1016/j.biocon.2019.05.053.

[ece371139-bib-0015] Guisan, A. , and W. Thuiller . 2005. “Predicting Species Distribution: Offering More Than Simple Habitat Models.” Ecology Letters 8, no. 9: 993–1009. 10.1111/j.1461-0248.2005.00792.x. 34517687

[ece371139-bib-0016] Guisande, C. , and J. Lobo . 2023. “*KnowBR: Discriminating Well Surveyed Spatial Units From Exhaustive Biodiversity Databases*.” *R package version 2.2* [Computer software]. https://CRAN.R‐project.org/package=KnowBR.

[ece371139-bib-0017] Hardy, H. , L. Livermore , P. Kersey , K. Norris , and V. Smith . 2023. “Understanding the Users and Uses of UK Natural History Collections.” Research Ideas & Outcomes 9: e113378. 10.3897/rio.9.e113378.

[ece371139-bib-0018] Heberling, J. M. , J. T. Miller , D. Noesgaard , S. B. Weingart , and D. Schigel . 2021. “Data Integration Enables Global Biodiversity Synthesis.” Proceedings of the National Academy of Sciences 118, no. 6: e2018093118. 10.1073/pnas.2018093118. PMC8017944 33526679

[ece371139-bib-0019] Hopkins, G. W. , and R. P. Freckleton . 2002. “Declines in the Numbers of Amateur and Professional Taxonomists: Implications for Conservation.” Animal Conservation 5, no. 3: 245–249. 10.1017/S1367943002002299.

[ece371139-bib-0020] Hortal, J. , F. De Bello , J. A. F. Diniz‐Filho , T. M. Lewinsohn , J. M. Lobo , and R. J. Ladle . 2015. “Seven Shortfalls That Beset Large‐Scale Knowledge of Biodiversity.” Annual Review of Ecology, Evolution, and Systematics 46, no. 1: 523–549. 10.1146/annurev-ecolsys-112414-054400.

[ece371139-bib-0021] Hortal, J. , A. Jiménez‐Valverde , J. F. Gómez , J. M. Lobo , and A. Baselga . 2008. “Historical Bias in Biodiversity Inventories Affects the Observed Environmental Niche of the Species.” Oikos 117, no. 6: 847–858. 10.1111/j.0030-1299.2008.16434.x.

[ece371139-bib-0022] Hughes, A. C. , M. C. Orr , K. Ma , et al. 2021. “Sampling Biases Shape Our View of the Natural World.” Ecography 44, no. 9: 1259–1269. 10.1111/ecog.05926.

[ece371139-bib-0023] Hutchings, P. 2021. “Potential Loss of Biodiversity and the Critical Importance of Taxonomy—An Australian Perspective.” In Advances in Marine Biology, vol. 88, 3–16. Elsevier. 10.1016/S0065-2881(21)00015-8. 34119045

[ece371139-bib-0024] Iannella, M. , P. D'Alessandro , and M. Biondi . 2019. “Entomological Knowledge in Madagascar by GBIF Datasets: Estimates on the Coverage and Possible Biases (Insecta).” Fragmenta Entomologica 51, no. 1: 1–10. 10.4081/fe.2019.329.

[ece371139-bib-0025] Importance of Taxonomy . 1946. “Importance of Taxonomy.” Nature 158: 105–106.

[ece371139-bib-0026] International Monetary Fund . 2024. “Report for Selected Countries and Subjects.” World Economic Outlook Databas. https://www.imf.org/en/Publications/WEO/weo‐database/2024/April/weo‐report.

[ece371139-bib-0027] Işik, K. 2011. “Rare and Endemic Species: Why Are They Prone to Extinction?” Turkish Journal of Botany 35: 411–417. 10.3906/bot-1012-90.

[ece371139-bib-0028] Lagomarsino, L. P. , and L. A. Frost . 2020. “The Central Role of Taxonomy in the Study of Neotropical Biodiversity.” Annals of the Missouri Botanical Garden 105, no. 3: 405–421. 10.3417/2020601.

[ece371139-bib-0029] Lobo, J. M. , J. Hortal , J. L. Yela , et al. 2018. “KnowBR: An Application to Map the Geographical Variation of Survey Effort and Identify Well‐Surveyed Areas From Biodiversity Databases.” Ecological Indicators 91: 241–248. 10.1016/j.ecolind.2018.03.077.

[ece371139-bib-0030] Lomolino, M. V. 2004. “Conservation Biogeography.” In Frontiers of Biogeography: New Directions in the Geography of Nature, edited by L. R. Heaney and M. V. Lomolino , 293–296. Sinauer Associates.

[ece371139-bib-0031] Massey, F. J. 1951. “The Kolmogorov‐Smirnov Test for Goodness of Fit.” Journal of the American Statistical Association 46, no. 253: 68–78. 10.2307/2280095.

[ece371139-bib-0032] Newbold, T. 2010. “Applications and Limitations of Museum Data for Conservation and Ecology, With Particular Attention to Species Distribution Models.” Progress in Physical Geography: Earth and Environment 34, no. 1: 3–22. 10.1177/0309133309355630.

[ece371139-bib-0033] Noriega, J. A. , J. Hortal , F. M. Azcárate , et al. 2018. “Research Trends in Ecosystem Services Provided by Insects.” Basic and Applied Ecology 26: 8–23. 10.1016/j.baae.2017.09.006.

[ece371139-bib-0034] Oliver, R. Y. , C. Meyer , A. Ranipeta , K. Winner , and W. Jetz . 2021. “Global and National Trends, Gaps, and Opportunities in Documenting and Monitoring Species Distributions.” PLoS Biology 19, no. 8: e3001336. 10.1371/journal.pbio.3001336. 34383738 PMC8360587

[ece371139-bib-0035] Olson, D. M. , E. Dinerstein , E. D. Wikramanayake , et al. 2001. “Terrestrial Ecoregions of the World: A New Map of Life on Earth.” Bioscience 51, no. 11: 933. 10.1641/0006-3568(2001)051[0933:TEOTWA]2.0.CO;2.

[ece371139-bib-0036] Popov, D. , P. Roychoudhury , H. Hardy , L. Livermore , and K. Norris . 2021. “The Value of Digitising Natural History Collections.” Research Ideas & Outcomes 7: e78844. 10.3897/rio.7.e78844.

[ece371139-bib-0037] Posit Team . 2024. “*RStudio: Integrated Development Environment for R* [Computer Software].” Posit Software, PBC. http://www.posit.co/.

[ece371139-bib-0038] Prudic, K. , E. Zylstra , N. Melkonoff , R. Laura , and R. Hutchinson . 2023. “Community Scientists Produce Open Data for Understanding Insects and Climate Change.” Current Opinion in Insect Science 59: 101081. 10.1016/j.cois.2023.101081. 37393063

[ece371139-bib-0039] QGIS . 2021. “*QGIS 3.22.9‐Białowieża* (Version 3.22.9‐Białowieża) [Computer Software].”

[ece371139-bib-0040] R Core Team . 2022. “R: A Language and Environment for Statistical Computing.” *R Foundation for Statistical Computing* [Computer software]. https://www.R‐project.org/.

[ece371139-bib-0041] Revelle, W. 2024. “*psych: Procedures for Psychological, Psychometric, and Personality Research* [Computer software].” Northwestern University. https://CRAN.R‐project.org/package=psych.

[ece371139-bib-0042] Rocha‐Ortega, M. , P. Rodriguez , and A. Córdoba‐Aguilar . 2021. “Geographical, Temporal and Taxonomic Biases in Insect GBIF Data on Biodiversity and Extinction.” Ecological Entomology 46, no. 4: 718–728. 10.1111/een.13027.

[ece371139-bib-0043] Romo, H. , E. García‐Barros , and J. M. Lobo . 2006. “Identifying Recorder‐Induced Geographic Bias in an Iberian Butterfly Database.” Ecography 29, no. 6: 873–885. 10.1111/j.2006.0906-7590.04680.x.

[ece371139-bib-0044] Ronquillo, C. 2023. “*cRonFer/Inv.Completeness‐Env.Space* [R].” https://github.com/cRonFer/Inv.Completeness‐Env.Space.

[ece371139-bib-0045] Ronquillo, C. , F. Alves‐Martins , V. Mazimpaka , et al. 2020. “Assessing Spatial and Temporal Biases and Gaps in the Publicly Available Distributional Information of Iberian Mosses.” Biodiversity Data Journal 8: e53474. 10.3897/BDJ.8.e53474. 33005091 PMC7508938

[ece371139-bib-0046] Ronquillo, C. , J. Stropp , and J. Hortal . 2024. “OCCUR Shiny Application: A User‐Friendly Guide for Curating Species Occurrence Records.” Methods in Ecology and Evolution 15, no. 5: 816–823. 10.1111/2041-210X.14271.

[ece371139-bib-0047] Ronquillo, C. , J. Stropp , N. G. Medina , and J. Hortal . 2023. “Exploring the Impact of Data Curation Criteria on the Observed Geographical Distribution of Mosses.” Ecology and Evolution 13, no. 12: e10786. 10.1002/ece3.10786. 38053793 PMC10694387

[ece371139-bib-0048] Sánchez‐Fernández, D. , R. Fox , R. L. H. Dennis , and J. M. Lobo . 2021. “How Complete Are Insect Inventories? An Assessment of the British Butterfly Database Highlighting the Influence of Dynamic Distribution Shifts on Sampling Completeness.” Biodiversity and Conservation 30, no. 3: 889–902. 10.1007/s10531-021-02122-w.

[ece371139-bib-0049] Sánchez‐Fernández, D. , J. M. Lobo , P. Abellán , I. Ribera , and A. Millán . 2008. “Bias in Freshwater Biodiversity Sampling: The Case of Iberian Water Beetles.” Diversity and Distributions 14, no. 5: 754–762. 10.1111/j.1472-4642.2008.00474.x.

[ece371139-bib-0050] Sánchez‐Fernández, D. , J. L. Yela , R. Acosta , et al. 2022. “Are Patterns of Sampling Effort and Completeness of Inventories Congruent? A Test Using Databases for Five Insect Taxa in the Iberian Peninsula.” Insect Conservation and Diversity 15, no. 4: 406–415. 10.1111/icad.12566.

[ece371139-bib-0051] Schoener, T. W. 1970. “Nonsynchronous Spatial Overlap of Lizards in Patchy Habitats.” Ecology 51, no. 3: 408–418. 10.2307/1935376.

[ece371139-bib-0052] Shirey, V. , M. W. Belitz , V. Barve , and R. Guralnick . 2021. “A Complete Inventory of North American Butterfly Occurrence Data: Narrowing Data Gaps, but Increasing Bias.” Ecography 44, no. 4: 537–547. 10.1111/ecog.05396.

[ece371139-bib-0053] Shirey, V. , S. Seppälä , V. Branco , and P. Cardoso . 2019. “Current GBIF Occurrence Data Demonstrates Both Promise and Limitations for Potential Red Listing of Spiders.” Biodiversity Data Journal 7: e47369. 10.3897/BDJ.7.e47369. 31885463 PMC6933025

[ece371139-bib-0054] Siddig, A. A. H. 2019. “Why Is Biodiversity Data‐Deficiency an Ongoing Conservation Dilemma in Africa?” Journal for Nature Conservation 50: 125719. 10.1016/j.jnc.2019.125719.

[ece371139-bib-0055] Smith, B. E. , M. K. Johnston , and R. Lücking . 2016. “From GenBank to GBIF: Phylogeny‐Based Predictive Niche Modeling Tests Accuracy of Taxonomic Identifications in Large Occurrence Data Repositories.” PLoS One 11, no. 3: e0151232. 10.1371/journal.pone.0151232. 26967999 PMC4788202

[ece371139-bib-0056] Soberón, J. , L. Arriaga , and L. Lara . 2002. “Issues of Quality Control in Large, Mixed‐Origin Entomological Databases.” In Towards a Global Biological Information Infrastructure.Pdf, edited by H. Saarenmaa and E. S. Nielsen , 15–22. European Environment Agency. https://www.eea.europa.eu/publications/technical_report_2001_70.

[ece371139-bib-0057] Soberón, J. , and T. Peterson . 2004. “Biodiversity Informatics: Managing and Applying Primary Biodiversity Data.” Philosophical Transactions of the Royal Society of London. Series B: Biological Sciences 359, no. 1444: 689–698. 10.1098/rstb.2003.1439. 15253354 PMC1693343

[ece371139-bib-0058] Sobral‐Souza, T. , J. Stropp , J. P. Santos , et al. 2021. “Knowledge Gaps Hamper Understanding the Relationship Between Fragmentation and Biodiversity Loss: The Case of Atlantic Forest Fruit‐Feeding Butterflies.” PeerJ 9: e11673. 10.7717/peerj.11673. 34239779 PMC8237826

[ece371139-bib-0059] Spector, S. 2006. “Scarabaeine Dung Beetles (Coleoptera: Scarabaeidae: Scarabaeinae): An Invertebrate Focal Taxon for Biodiversity Research and Conservation.” Coleopterists Bulletin 60: 71–83.

[ece371139-bib-0060] Sporbert, M. , H. Bruelheide , G. Seidler , et al. 2019. “Assessing Sampling Coverage of Species Distribution in Biodiversity Databases.” Journal of Vegetation Science 30, no. 4: 620–632. 10.1111/jvs.12763.

[ece371139-bib-0061] Stropp, J. , R. J. Ladle , A. C. Malhado , et al. 2016. “Mapping Ignorance: 300 Years of Collecting Flowering Plants in Africa.” Global Ecology and Biogeography 25, no. 9: 1085–1096. 10.1111/geb.12468.

[ece371139-bib-0062] Takano, H. 2025. “A systematic revision of the Afrotropical members of the dung beetle genus Catharsius Hope, 1837 (Coleoptera: Scarabaeidae).” [Manuscript in Preparation]. African Natural History Research Trust.

[ece371139-bib-0063] Troia, M. J. , and R. A. McManamay . 2016. “Filling in the GAPS : Evaluating Completeness and Coverage of Open‐Access Biodiversity Databases in the United States.” Ecology and Evolution 6, no. 14: 4654–4669. 10.1002/ece3.2225. 27547303 PMC4979697

[ece371139-bib-0064] Troudet, J. , P. Grandcolas , A. Blin , R. Vignes‐Lebbe , and F. Legendre . 2017. “Taxonomic Bias in Biodiversity Data and Societal Preferences.” Scientific Reports 7, no. 1: 9132. 10.1038/s41598-017-09084-6. 28831097 PMC5567328

[ece371139-bib-0065] United Nations Statistics Division . n.d. “UNSD—Methodology. Standard Country or Area Codes for Statistical Use (M49).” https://unstats.un.org/unsd/methodology/m49/.

[ece371139-bib-0066] Wägele, H. , A. Klussmann‐Kolb , M. Kuhlmann , et al. 2011. “The Taxonomist—An Endangered Race. A Practical Proposal for Its Survival.” Frontiers in Zoology 8, no. 1: 25. 10.1186/1742-9994-8-25. 22029904 PMC3210083

[ece371139-bib-0067] Wagner, D. L. , E. M. Grames , M. L. Forister , M. R. Berenbaum , and D. Stopak . 2021. “Insect Decline in the Anthropocene: Death by a Thousand Cuts.” Proceedings of the National Academy of Sciences 118, no. 2: e2023989118. 10.1073/pnas.2023989118. PMC7812858 33431573

[ece371139-bib-0068] World Wildlife Fund . 2012. “Publications: Terrestrial Ecoregions of the World.” World Wildlife Fund. https://www.worldwildlife.org/publications/terrestrial‐ecoregions‐of‐the‐world.

[ece371139-bib-0069] WorldClim . 2020, 2022. “*Bioclimatic variables.”* https://www.worldclim.org/data/bioclim.html.

[ece371139-bib-0070] Wüest, R. O. , N. E. Zimmermann , D. Zurell , et al. 2020. “Macroecology in the Age of Big Data—Where to Go From Here?” Journal of Biogeography 47, no. 1: 1–12. 10.1111/jbi.13633.

[ece371139-bib-0071] Yesson, C. , P. W. Brewer , T. Sutton , et al. 2007. “How Global Is the Global Biodiversity Information Facility?” PLoS One 2, no. 11: e1124. 10.1371/journal.pone.0001124. 17987112 PMC2043490

